# Supplementary material for: Multi-locus phylogenetic network analysis of Ampelomyces mycoparasites isolated from diverse powdery mildews in Australia and the generation of two de novo genome assemblies
Source: PLoS One. 2025 Dec 4;20(12):e0322842. doi: 10.1371/journal.pone.0322842 (PMC12677482; doi:10.1371/journal.pone.0322842)
Supplement: S2 Fig — Clustal W software was used for the eukNR alignment and then Bayesian inference was used to infer the tree. The four Ampelomyces strains that have whole genome sequencing assemblies are indicated in bold. (PPTX) [file pone.0322842.s002.pptx]

## Slide 1
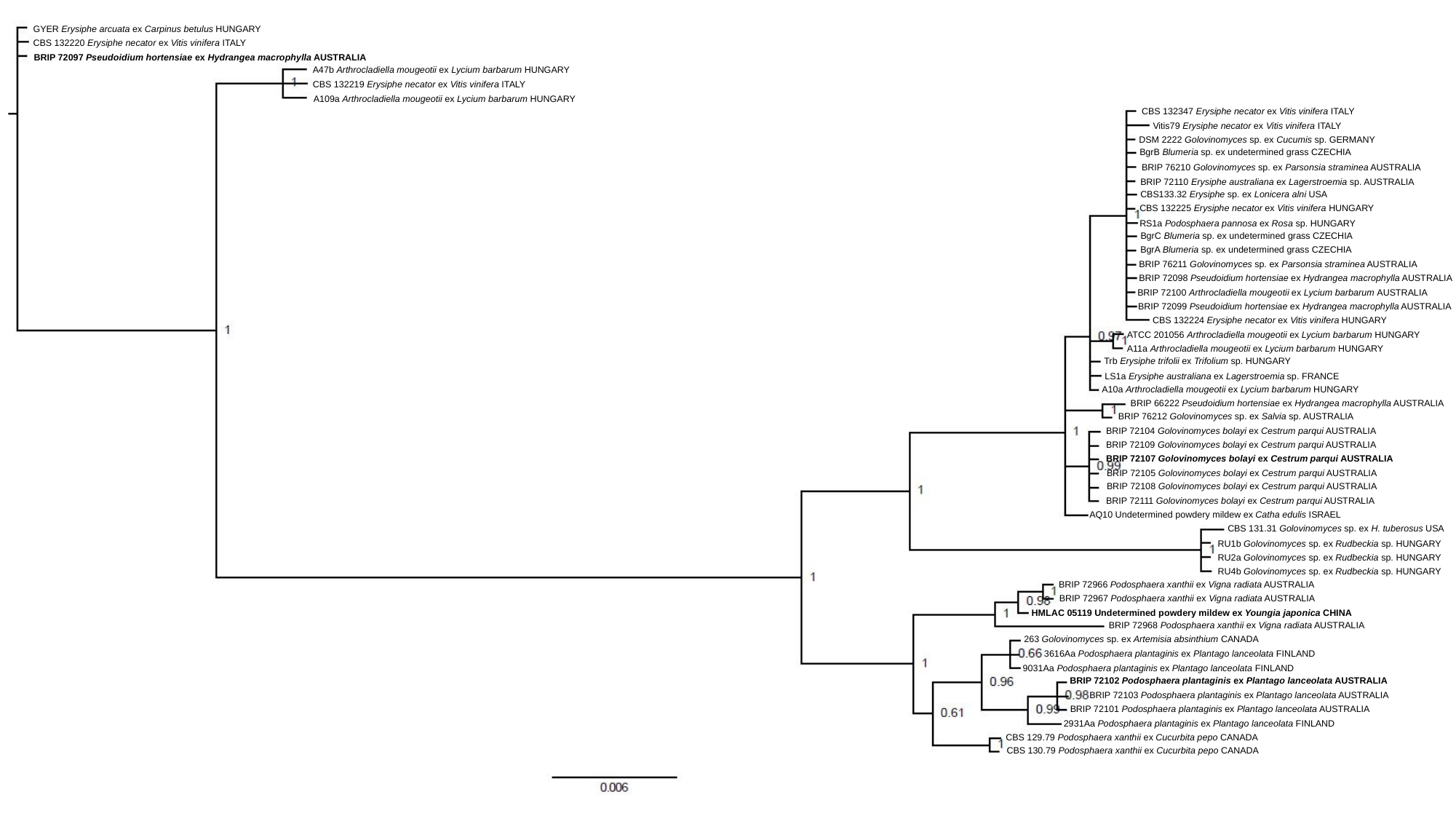

GYER Erysiphe arcuata ex Carpinus betulus HUNGARY
CBS 132220 Erysiphe necator ex Vitis vinifera ITALY
BRIP 72097 Pseudoidium hortensiae ex Hydrangea macrophylla AUSTRALIA
A47b Arthrocladiella mougeotii ex Lycium barbarum HUNGARY
CBS 132219 Erysiphe necator ex Vitis vinifera ITALY
A109a Arthrocladiella mougeotii ex Lycium barbarum HUNGARY
CBS 132347 Erysiphe necator ex Vitis vinifera ITALY
Vitis79 Erysiphe necator ex Vitis vinifera ITALY
DSM 2222 Golovinomyces sp. ex Cucumis sp. GERMANY
BgrB Blumeria sp. ex undetermined grass CZECHIA
BRIP 76210 Golovinomyces sp. ex Parsonsia straminea AUSTRALIA
BRIP 72110 Erysiphe australiana ex Lagerstroemia sp. AUSTRALIA
CBS133.32 Erysiphe sp. ex Lonicera alni USA
CBS 132225 Erysiphe necator ex Vitis vinifera HUNGARY
RS1a Podosphaera pannosa ex Rosa sp. HUNGARY
BgrC Blumeria sp. ex undetermined grass CZECHIA
BgrA Blumeria sp. ex undetermined grass CZECHIA
BRIP 76211 Golovinomyces sp. ex Parsonsia straminea AUSTRALIA
BRIP 72098 Pseudoidium hortensiae ex Hydrangea macrophylla AUSTRALIA
BRIP 72100 Arthrocladiella mougeotii ex Lycium barbarum AUSTRALIA
BRIP 72099 Pseudoidium hortensiae ex Hydrangea macrophylla AUSTRALIA
CBS 132224 Erysiphe necator ex Vitis vinifera HUNGARY
ATCC 201056 Arthrocladiella mougeotii ex Lycium barbarum HUNGARY
A11a Arthrocladiella mougeotii ex Lycium barbarum HUNGARY
Trb Erysiphe trifolii ex Trifolium sp. HUNGARY
LS1a Erysiphe australiana ex Lagerstroemia sp. FRANCE
A10a Arthrocladiella mougeotii ex Lycium barbarum HUNGARY
BRIP 66222 Pseudoidium hortensiae ex Hydrangea macrophylla AUSTRALIA
BRIP 76212 Golovinomyces sp. ex Salvia sp. AUSTRALIA
BRIP 72104 Golovinomyces bolayi ex Cestrum parqui AUSTRALIA
BRIP 72109 Golovinomyces bolayi ex Cestrum parqui AUSTRALIA
BRIP 72107 Golovinomyces bolayi ex Cestrum parqui AUSTRALIA
BRIP 72105 Golovinomyces bolayi ex Cestrum parqui AUSTRALIA
BRIP 72108 Golovinomyces bolayi ex Cestrum parqui AUSTRALIA
BRIP 72111 Golovinomyces bolayi ex Cestrum parqui AUSTRALIA
AQ10 Undetermined powdery mildew ex Catha edulis ISRAEL
CBS 131.31 Golovinomyces sp. ex H. tuberosus USA
RU1b Golovinomyces sp. ex Rudbeckia sp. HUNGARY
RU2a Golovinomyces sp. ex Rudbeckia sp. HUNGARY
RU4b Golovinomyces sp. ex Rudbeckia sp. HUNGARY
BRIP 72966 Podosphaera xanthii ex Vigna radiata AUSTRALIA
BRIP 72967 Podosphaera xanthii ex Vigna radiata AUSTRALIA
HMLAC 05119 Undetermined powdery mildew ex Youngia japonica CHINA
BRIP 72968 Podosphaera xanthii ex Vigna radiata AUSTRALIA
263 Golovinomyces sp. ex Artemisia absinthium CANADA
3616Aa Podosphaera plantaginis ex Plantago lanceolata FINLAND
9031Aa Podosphaera plantaginis ex Plantago lanceolata FINLAND
BRIP 72102 Podosphaera plantaginis ex Plantago lanceolata AUSTRALIA
BRIP 72103 Podosphaera plantaginis ex Plantago lanceolata AUSTRALIA
BRIP 72101 Podosphaera plantaginis ex Plantago lanceolata AUSTRALIA
2931Aa Podosphaera plantaginis ex Plantago lanceolata FINLAND
CBS 129.79 Podosphaera xanthii ex Cucurbita pepo CANADA
CBS 130.79 Podosphaera xanthii ex Cucurbita pepo CANADA
