# Supplementary material for: Multi-locus phylogenetic network analysis of Ampelomyces mycoparasites isolated from diverse powdery mildews in Australia and the generation of two de novo genome assemblies
Source: PLoS One. 2025 Dec 4;20(12):e0322842. doi: 10.1371/journal.pone.0322842 (PMC12677482; doi:10.1371/journal.pone.0322842)
Supplement: S3 Fig — Vertical blue lines indicate the GC cut-off points selected by OcculterCut (43) to classify genome segments into distinct AT-rich and GC-balanced regions. The percent values on the left and right sides of the graphs indicate the percentage of the genome classified as AT-rich and GC-balanced, respectively. (PPTX) [file pone.0322842.s003.pptx]

## Slide 1
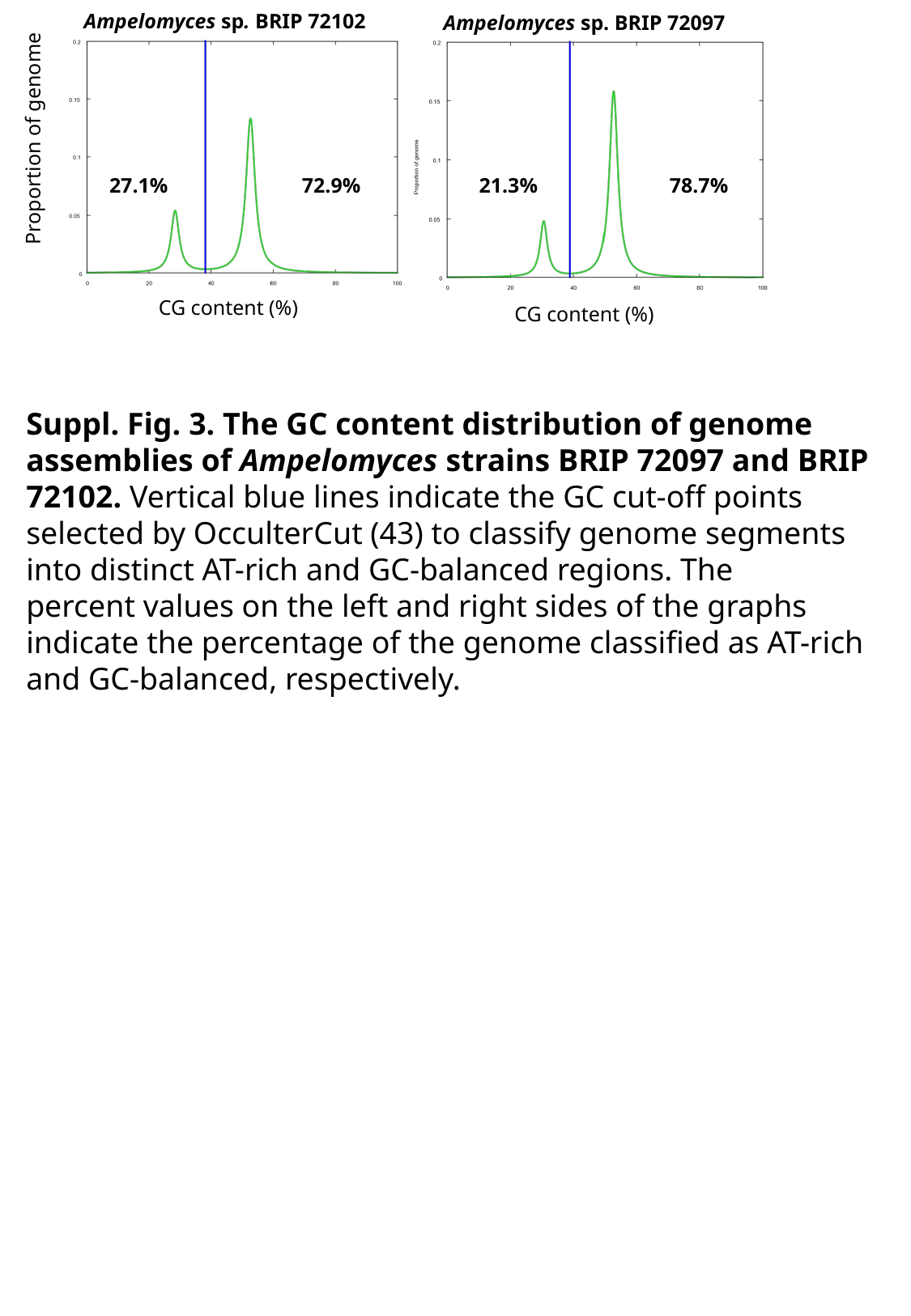

Ampelomyces sp. BRIP 72102
 Ampelomyces sp. BRIP 72097
Proportion of genome
27.1%
78.7%
72.9%
21.3%
CG content (%)
CG content (%)
Suppl. Fig. 3. The GC content distribution of genome assemblies of Ampelomyces strains BRIP 72097 and BRIP 72102. Vertical blue lines indicate the GC cut-off points selected by OcculterCut (43) to classify genome segments into distinct AT-rich and GC-balanced regions. The percent values on the left and right sides of the graphs indicate the percentage of the genome classified as AT-rich and GC-balanced, respectively.
