# Supplementary material for: Multi-locus phylogenetic network analysis of Ampelomyces mycoparasites isolated from diverse powdery mildews in Australia and the generation of two de novo genome assemblies
Source: PLoS One. 2025 Dec 4;20(12):e0322842. doi: 10.1371/journal.pone.0322842 (PMC12677482; doi:10.1371/journal.pone.0322842)
Supplement: Attachment — Submitted filename: Ampelomyces_Australia_RESPONSE TO COMMENTS_2nd revision.docx [file pone.0322842.s007.docx]

**Professor Kandasamy Ulaganathan**

**Centre for Plant Molecular Biology, Osmania University, Hyderabad, India**

**Academic Editor, PLoS ONE**

Dear Professor Ulaganathan,

Thank you for your comments on our work and judging it as suitable for publication in PLoS ONE pending a 2^nd^ round of revision.

Please find below our responses and reactions to the comments from Reviewers #1 and #2. Line (L) numbers in our responses refer to the file uploaded as ‘2nd Revision of Manuscript with Tracked Changes’.

In addition to minor changes done in line with the 2^nd^ Reviewer’s comments (as detailed below), we have also done a few cosmetic changes in the manuscript – all shown with tracked changes.

We would like to sincerely thank both Reviewers for their time spent on our manuscript.

**The file uploaded as ‘Manuscript’ was generated by accepting all tracked changes shown in ‘2nd Revision of Manuscript with Tracked Changes’.**

Thank you for considering this 2^nd^ revision. We do hope that the current version is suitable for publication in PLoS ONE.

Best regards,

Prof Levente Kiss

corresponding author for this submission

**Response to Reviewer #1**
>COMMENT:

*The article "Multi-locus phylogenetic network analysis of Ampelomyces mycoparasites isolated from diverse powdery mildews in Australia and the generation of two de novo genome assemblies" provide important detailed analysis on Ampelomyces from diverse powdery mildews in Australia. It is an great work that offers valuable contribution to the scientific community in this field. Minor revision is suggested since I agree with reviewer 2 that "Figure 3 also needs to be rooted or drawn more explicitly as an unrooted tree like Fig 4."*

RESPONSE: Thank you for your kind words. Concerning the three unrooted trees (Figure 3 – ITS tree; and Suppl. Figs. 1 and 2 – the act and the eukNR trees, respectively), these have to remain unrooted because of the difficulty of finding the same outgroup for all these three loci. This was explained in our previous response to Reviewer #2.

To highlight the unrooted nature of these three trees, the captions of all the three figures start with ‘Unrooted tree of …’.

We have experimented with replacing these three figures with network-like and circular trees. However, one of the values of these trees is to display all the details (designations, mycohosts, host plants, and countries of origin) of the mycoparasitic strains included in the analysis, and this can only be achieved in the current format of Figure 3 and Suppl. Figs. 1 and 2. The phylogenetic network (Figure 4) could not display all the information that is available on the unrooted trees.

This is the rationale for presenting these three figures in their current format.

**Response to Reviewer #2**

>COMMENT:

*The authors have made a substantial effort to address the previous comments. I think the paper is clearer as a result. I only have a couple of minor residual comments/questions about the BUSCO analysis.*

RESPONSE: Thank you for your positive feedback.

>COMMENT:

*1. Did the authors really use BUSCO v1.2? I did not notice this the first time. BUSCO is now up to v6 and the more recent versions are much more consistent, so should be used preferentially. It does not matter too much for this paper, but the authors mention ongoing work, so they really should switch to at least v5 for that.*

RESPONSE AND REACTION: Thank you for spotting this typo. We used BUSCO v5.8.0 and corrected the typo in L343.

>COMMENT:

*2. The proteome analysis uses a different orthdb dataset to the genome analysis. Please use the same for both. It is hard to judge the actual quality of the annotation if the stats relate to a different dataset.*

RESPONSE AND REACTION: Thank you for spotting this issue. The proteome BUSCO was run again with *dothideomycetes_odb10* and the results were updated in the manuscript (L422-425 and L444-446).
